# Targeting Myeloid Cells to the Brain Using Non-Myeloablative Conditioning

**DOI:** 10.1371/journal.pone.0080260

**Published:** 2013-11-07

**Authors:** Chotima Böttcher, Francisco Fernández-Klett, Nadine Gladow, Simone Rolfes, Josef Priller

**Affiliations:** 1 Department of Neuropsychiatry and Laboratory of Molecular Psychiatry, Charité Universitätsmedizin Berlin, Berlin, Germany; 2 Cluster of Excellence NeuroCure, Berlin, Germany; University of Frankfurt - University Hospital Frankfurt, Germany

## Abstract

Bone marrow-derived cells (BMDCs) are able to colonize the central nervous system (CNS) at sites of damage. This ability makes BMDCs an ideal cellular vehicle for transferring therapeutic genes/molecules to the CNS. However, conditioning is required for bone marrow-derived myeloid cells to engraft in the brain, which so far has been achieved by total body irradiation (TBI) and by chemotherapy (e.g. busulfan treatment). Unfortunately, both regimens massively disturb the host’s hematopoietic compartment. Here, we established a conditioning protocol to target myeloid cells to sites of brain damage in mice using non-myeloablative focal head irradiation (HI). This treatment was associated with comparatively low inflammatory responses in the CNS despite cranial radiation doses which are identical to TBI, as revealed by gene expression analysis of cytokines/chemokines such as CCL2, CXCL10, TNF-α and CCL5. HI prior to bone marrow transplantation resulted in much lower levels of blood chimerism defined as the percentage of donor-derived cells in peripheral blood (< 5%) compared with TBI (> 95%) or busulfan treatment (>50%). Nevertheless, HI effectively recruited myeloid cells to the area of motoneuron degeneration in the brainstem within 7 days after facial nerve axotomy. In contrast, no donor-derived cells were detected in the lesioned facial nucleus of busulfan-treated animals up to 2 weeks after transplantation. Our findings suggest that myeloid cells can be targeted to sites of brain damage even in the presence of very low levels of peripheral blood chimerism. We established a novel non-myeloablative conditioning protocol with minimal disturbance of the host’s hematopoietic system for targeting BMDCs specifically to areas of pathology in the brain.

## Introduction

Microglia are the key immune effector cells of the central nervous system (CNS), mediating local inflammatory and innate immune responses. This CNS cell population has recently been shown to derive from immature yolk sac macrophages that infiltrate the brain during early embryogenesis [1-3]. Thus, microglia are ontogenetically distinct from hematopoietic stem cell (HSC)-derived tissue macrophages that originate from the bone marrow continuously throughout adult life. In line with these findings, no engraftment of myeloid cells was observed in the CNS of parabiotic mice, in which blood from two different animals is chronically shunted, suggesting that circulating hematopoietic progenitors and circulating monocytes do not significantly contribute to microglia homeostasis after birth [1-5].

However, bone marrow-derived cells (BMDCs) are able to colonize the adult CNS under certain conditions. Transplantation of genetically labelled BMDCs into total body-irradiated hosts has demonstrated that circulating myeloid cells engraft in the CNS and contribute to the pool of brain macrophages, both in the absence and during overt brain pathology [6-21]. Irradiation-induced changes in the CNS, as well as the introduction of hematopoietic stem/progenitor cells into the circulation have been suggested as necessary conditions for the recruitment of myeloid cells into the brain [1,4,21]. 

In line with their different origin, bone marrow-derived myeloid cells and microglia appear to exert differential functions in the CNS. In a mouse model of Alzheimer’s disease, myeloid cells were able to phagocytose β-amyloid, whereas resident microglia appeared to be rather ineffective in this task [13,14]. Similarly, BMDCs were found to attenuate or even arrest pathology in mouse models of neuropsychiatric disorders including Rett syndrome, amyotrophic lateral sclerosis, Krabbe’s disease and Parkinson’s disease [19,20,22,23]. Hence, myeloid cells have a tremendous therapeutic potential for neurological and psychiatric diseases. However, establishing a clinical conditioning regimen remains a challenge. Although total body irradiation (TBI) is an effective conditioning protocol to target the myeloid cells to the brain, this myeloablative treatment induces massive CNS inflammation and disturbance of the host’s hematopoietic system [21,24]. Recently, conditioning with the alkylating chemotherapeutic agent, busulfan, has been suggested as an alternative [24]. Indeed, myeloablation with busulfan is being used in the clinical setting [25]. However, myeloid cell engraftment at sites of CNS damage after busulfan conditioning in mice was either absent [26] or dramatically reduced compared to irradiation [24]. 

Here, we established a protocol for CNS conditioning using focal head irradiation (HI) that avoids myeloablation and minimally disturbs the host’s hematopoietic system. Regardless of the low presence of donor-derived cells in the peripheral circulation, BMDCs rapidly and selectively engrafted at sites of neurodegeneration. This conditioning regimen may serve as an alternative protocol for targeting myeloid cells to the CNS with minimal impairment of the hematopoietic compartment. 

## Materials and Methods

### Mice

C57BL/6 wild type mice were purchased from Charles River (Sulzbach). C57BL/6 mice expressing the enhanced green fluorescent protein (GFP) under the control of β-actin promoter (*ACTβ-EGFP*) [27] were obtained from breeding facility of Charité. All recipient mice were 7-12 weeks old at the time of bone marrow transplantation (BMT).

All animal experiments were performed in strict accordance with national and international guidelines for the care and use of laboratory animals (Tierschutzgesetz der Bundesrepublik Deutschland, European directive 2010/63/EU, as well as GV-SOLAS and FELASA guidelines and recommendations for laboratory animal welfare). The experiments were specifically approved by the committee on the ethics of animal experiments of Berlin (Landesamt für Gesundheit und Soziales, Berlin, Germany, Permit Number: G0364/10). 

### Conditioning

Mice were anesthetized by subcutaneous injection of a mixture of ketamine (50 mg/kg) and xylazine (7.5 mg/kg) prior to either total body irradiation (TBI) or focal head irradiation (HI) with a single dose of 11Gy. Irradiation was performed using a Caesium^137^ source (Gammacell 40 Exactor, Theratronics). During HI, the body was protected from irradiation with lead bars (3 cm thick). Dosimetric studies revealed a shielding efficieny >90% (cumulative dose < 1 Gy in protected areas). Busulfan-treated animals received two intraperitoneal (i.p.) injections of 50 mg/kg busulfan (Busilvex®, Pierre Fabre Pharma) at 5 and 3 days before BMT.

### Bone marrow transplantation

After conditioning with either TBI or HI, wild type mice were intravenously (i.v.) injected with 2 x 10^7^ unsorted bone marrow cells from *ACTβ-EGFP* mice within 24 hr after irradiation. Animals were examined at 1, 2, 4 and 16 weeks post-transplantation. 

In the case of facial nerve axotomy (FNA), conditioning with HI was performed at 2 weeks and with busulfan at 5 and 3 days prior to BMT (i.v. injection of 2 x 10^7^ unsorted bone marrow cells from *ACTβ-EGFP* mice). FNA was performed 24 hr before BMT and animals received daily i.p. injections of 2 mg/kg rapamycin (Enzo Life Science) thereafter. Groups with HI or FNA alone served as controls. Animals were examined at 7 and 14 days post-transplantation. 

### Facial nerve axotomy

Facial nerve axotomy (FNA) was performed as described previously [6]. Briefly, mice were anesthetized by subcutaneous injection of a mixture of ketamine (50 mg/kg) and xylazine (7.5 mg/kg). The right facial nerve was transected at the stylomastoid foramen, resulting in ipsilateral whisker paresis. The left facial nerve served as control. 

### Immunohistochemistry

Mice were anesthetized and perfused transcardially with cold phosphate-buffered saline (PBS). Brains were dissected, post-fixed in 4% paraformaldehyde (PFA) and cryoprotected with 30% sucrose. Coronal brain sections (30 µm) were obtained on a cryostat. 

Sections were blocked at room temperature for 1 hr with 20% normal goat/donkey serum (Biozol) in Tris-buffered saline (TBS) containing 0.3% Triton X-100. After three washes in TBS, sections were incubated with primary antibodies diluted 1:200 [anti-Iba-1 (Wako), anti-F4/80 (Invitrogen) or anti-GFP (Nacalai Tesque, Invitrogen)] at 4°C overnight. After washing with TBS, sections were incubated with Alexa-conjugated secondary antibodies diluted 1:250 (Alexa 488-IgG and Alexa 594-IgG, Invitrogen) at room temperature for 3 hr. All antibodies were diluted in TBS containing 1% normal goat/donkey serum and 0.3% Triton X-100. Nuclei were counterstained with 4,6-diamidino-2-phenylindole diluted 1:10,000 (DAPI, Sigma). The immunostained sections were examined using a conventional fluorescence or laser-scanning confocal microscope (Leica TCS SP5, Leica Microsystems).

### Flow cytometric analysis

Spleens were excised and pushed through a 70-μm strainer, bone marrow cells were flushed from both femurs and tibias. All samples were collected in Dulbecco's PBS (Gibco) containing 2% fetal bovine serum (Biochrom) and were stored on ice during staining and analysis. Blood was collected from the inferior vena cava using a citrate-coated syringe. Red blood cells were lysed in Pharm Lyse^TM^ buffer (BD Biosciences).

Following Fc blocking, cells were stained with anti-CD115 (AFS98), Ly6C (AL-21), CD11b (M1/70), CD4 (RM4-5), CD8 (53-6.7), Ly6G (1A8) and CD19 (6D5). All antibodies were purchased from Biolegend. Fluorescence-activated cell sorting (FACS) was performed using a Canto II (Becton Dickinson). Forward- and side-scatter parameters were used for exclusion of doublets from analysis. Data were analyzed with the FlowJo software (TreeStar).

### Quantitative real-time PCR (qPCR)

Animals were anesthetized and perfused transcardially with cold PBS at 1, 2, 4 and 16 weeks after irradiation/BMT. Brains were dissected and immediately shock-frozen in liquid nitrogen. Total RNA was isolated from the brain using RNeasy Plus Mini kit (Qiagen). RNA (approximately 2 µg) was transcribed into cDNA using Amplitaq^®^ DNA Polymerase kit (Applied Biosystems, Roche). PCR reactions were carried out using the LightCycler FastStart DNA Master Kit (Roche Molecular Biochemicals) according to the manufacturer’s protocol. The following primer pairs were used: CCL5 (forward 5’-TGC CCA CGT CAA GGA GTA TTT-3’, reverse 5’-TCT CTG GGT TGG CAC ACA CTT-3’), CXCL10 (forward 5’-TGC TGG GTC TGA GTG GGA CT-3’, reverse 5’-CCC TAT GGC CCT CAT TCT CAC-3’), TNF-α (forward 5’-CAT CTT CTC AAA ATT CGA GTG ACA A-3’, reverse 5’-TGG GAG TAG ACA AGG TAC AAC CC-3’) and CCL2 (forward 5’-TCT GGG CCT GCT GTT CAC C-3’, reverse 5’-TTG GGA TCA TCT TGC TGG TG-3’). qPCR was performed using a LightCycler 2.0 (Roche).

### Statistical analysis

Results were analyzed with Prism 4.0 (GraphPad) and statistical differences were evaluated using a non-paired Student’s *t* test or one-way ANOVA with Posthoc Bonferroni correction. Significance was accepted for p<0.05. Data are shown as means ± SEM.

## Results

### Focal head irradiation induces delayed BMDC engraftment in the brain

**Figure 1:**
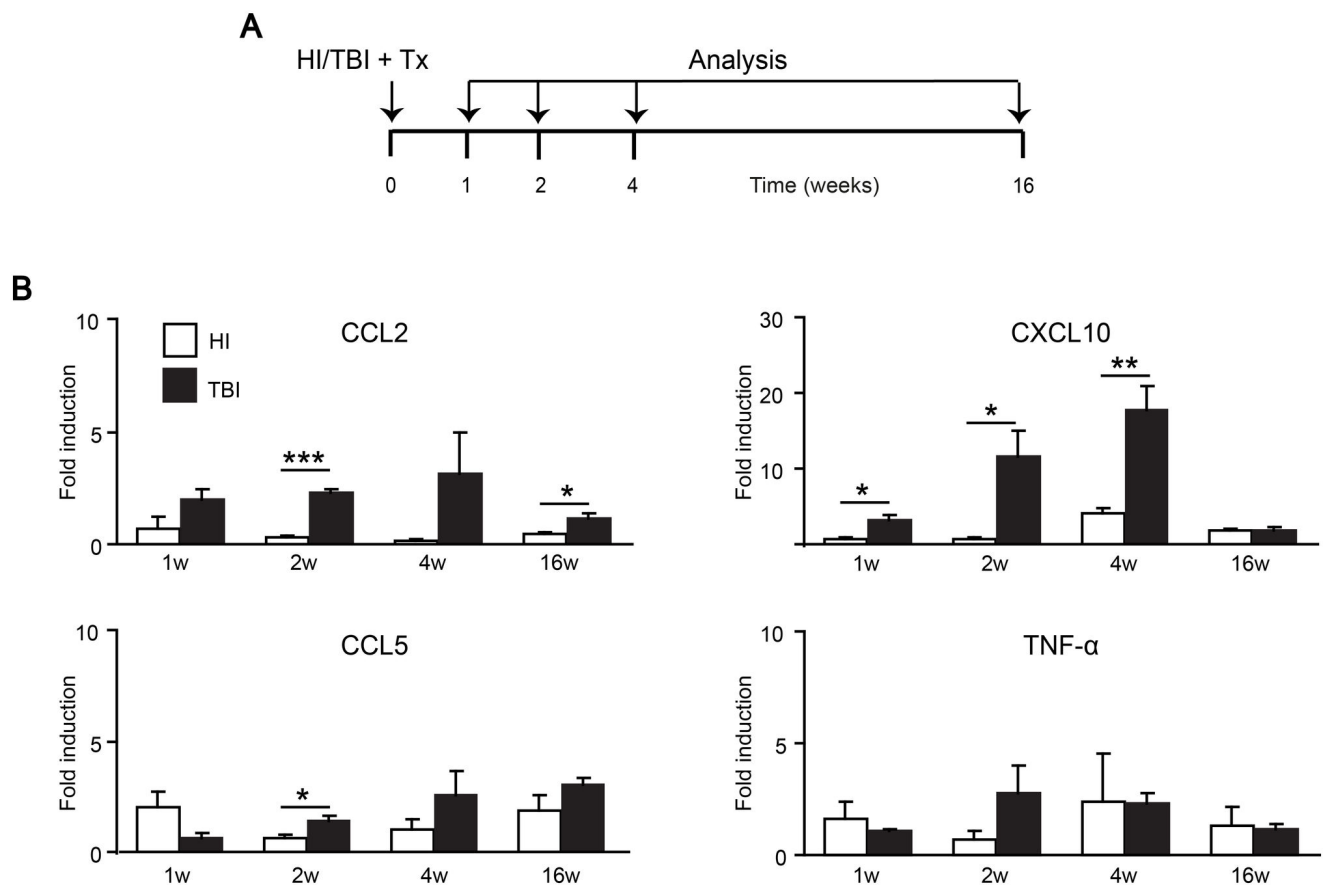
Gene expression profiles of cytokines and chemokines in the brain after HI and TBI. **A**) Overview of the experimental protocol. *HI*, *TBI* and *Tx* denote focal head irradiation, total body irradiation and bone marrow transplantation, respectively. **B**) Quantitative real-time PCR of CCL2, CXCL10, CCL5 and TNF-α mRNA expression in brains of HI (grey columns) and TBI (white columns) animals at 1, 2, 4 and 16 weeks after irradiation and BMT. The mRNA expression levels were normalized to GAPDH mRNA and compared to naïve mice (fold induction). Reduced cytokine/chemokine mRNA levels were observed in HI brains compared to the TBI paradigm. Data are means + SEM from 3-5 animals per group. Statistical significance is indicated by asterisks (*p<0.05; **p<0.01; ***p<0.001).

Irradiation has been described to be a necessary condition for BMDC engraftment in the brain [21]. We hypothesized that non-myeloablative conditioning using HI would induce CNS microenvironment changes that suffice to trigger the recruitment of circulating myeloid cells to the CNS in the absence of full hematopoietic reconstitution. To this end, we transplanted adult mice with unsorted GFP-expressing bone marrow cells following either HI or TBI (Figure 1A). Thereafter, we analyzed the induction of different cytokines and chemokines in the brain (Figure 1B). Irradiation-induced increases in gene expression of monocyte chemoattractant protein-1 (MCP-1 or CCL2), interferon gamma-induced protein 10 (IP-10 or CXCL10), regulated on activation, normal T cell expressed and secreted (RANTES or CCL5) and tumor necrosis factor (TNF)-α were observed in the brains of both TBI and HI mice (Figure 1B). However, the induction of CCL2 and CXCL10 mRNAs was strongly reduced in HI compared with TBI animals although the doses of radiation to the brain were identical (Figure 1B). CCL5 and TNF-α mRNAs were also reduced at 2 weeks after HI compared with TBI (Figure 1B). 

**Figure 2:**
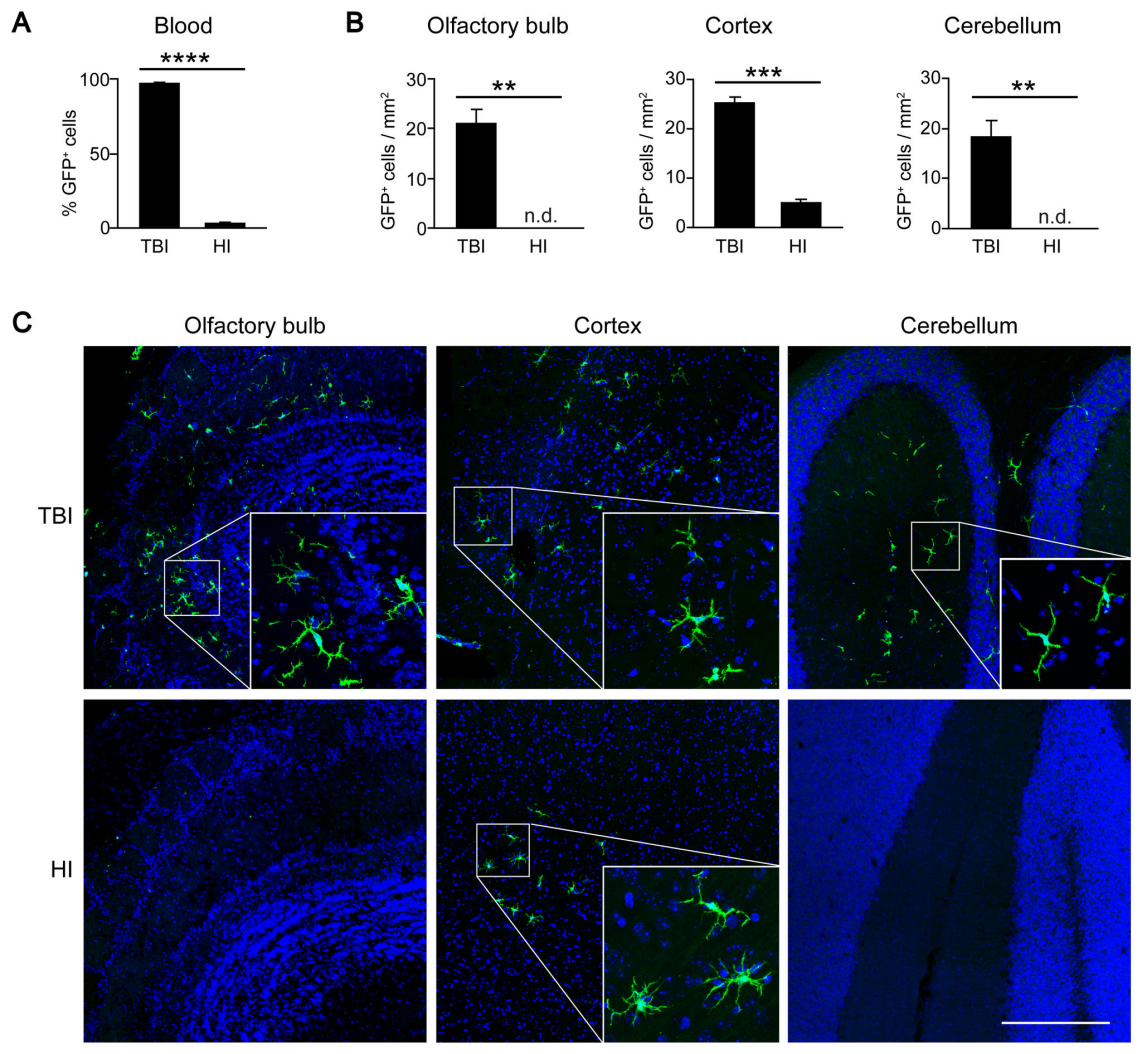
Delayed engraftment of BMDCs after HI. **A**) Flow cytometric analysis of GFP expression in peripheral blood leukocytes of HI and TBI chimeras. The level of chimerism was significantly lower in HI compared with TBI animals at 16 weeks after transplantation. Data are means + SEM from 3-5 animals per group. Statistical significance is indicated by the asterisk (****p<0.0001). **B**) Quantification of BMDC engraftment in the brains of HI and TBI chimeras. Data are expressed as GFP^+^ cells per area in three different brain regions (olfactory bulb, cortex and cerebellum) at 16 weeks after BMT. Data are means + SEM from 3-5 animals per group. n.d. = none detected. Statistical significance is indicated by asterisks (**p<0.01; ***p<0.001). **C**) Representative laser confocal microscopic images of ramified donor-derived GFP^+^ cells in the brains of HI and TBI animals at 16 weeks after BMT. Note that GFP^+^ cells were restricted to the cortex in the HI group, but distributed throughout the grey and white matter in TBI animals.

As expected, chimerism (determined as the percentage of GFP^+^CD45^+^ cells among all CD45^+^ cells) was significantly lower in peripheral blood of HI mice compared with TBI animals at 16 weeks post-BMT (TBI: 97 ± 0.6%, HI: 3 ± 0.3%; Figure 2A). When analyzing the brains of the chimeras, no donor-derived GFP^+^ cells were detected up to 12 weeks after BMT in HI animals, whereas TBI animals showed engraftment of ramified BMDCs in all brain regions (data not shown). At 16 weeks after BMT, clusters of donor-derived GFP^+^ cells appeared in the cortex of HI mice (Figures 2B,C). At this time point, olfactory bulb, cortex and cerebellum were populated by ramified GFP^+^ cells in the TBI group (Figures 2B,C). Quantitative analysis revealed reduced BMDC engraftment in the brains of HI mice compared with TBI animals (Figure 2B). The numbers of ramified GFP^+^ cells were 5 ± 1/ mm^2^ (TBI) versus 0/ mm^2^ (HI) in the olfactory bulb, 25 ± 1/ mm^2^ (TBI) versus 5 ± 1/ mm^2^ (HI) in the cortex, and 18 ± 3/ mm^2^ (TBI) versus 0/ mm^2^ (HI) in the cerebellum. These results suggest that low blood chimerism and reduced expression of chemoattractants like CCL2 in the brain result in reduced and delayed engraftment of BMDCs in the HI protocol.

### Enhanced recruitment of myeloid cells to sites of brain damage

**Figure 3:**
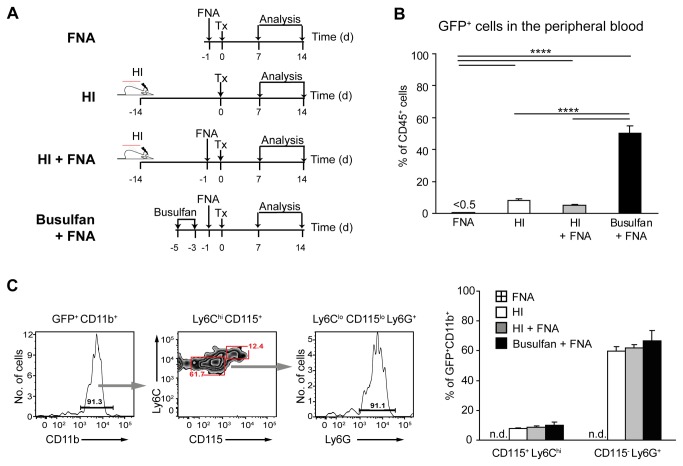
Blood chimerism in HI- and busulfan-conditioned mice with FNA. **A**) Overview of the experimental protocol. *HI*, *FNA* and *Tx* denote focal head irradiation, facial nerve axotomy and bone marrow transplantation, respectively. **B**) Flow cytometry of GFP expression in peripheral blood CD45^+^ cells at 2 weeks after BMT. The level of chimerism was significantly higher in the busulfan + FNA group compared with the HI + FNA group. FNA had no impact on blood chimerism. Data are means + SEM from 3-5 animals per group. Statistical significance is indicated by asterisks (****p<0.0001). **C**) Flow cytometric characterization of GFP-expressing cells in peripheral blood at 2 weeks after BMT. The vast majority of GFP^+^CD45^+^ cells express CD11b (>90%). Gating of this cell population reveals predominantly CD115^+^Ly6C^hi^Ly6G^-^ monocytes and CD115^-^Ly6C^+^Ly6G^+^ neutrophils. Data are means + SEM from 3-5 animals per group. n.d. = none detected. No statistical differences were observed between the groups.

We next tested whether HI in combination with neuronal damage accelerates the recruitment of BMDCs to the brain. To this end, we performed facial nerve axotomy in chimeric mice, which results in motoneuron degeneration in the absence of blood-brain barrier disruption [6]. Given that some potential precursors of brain macrophages, such as bone marrow-derived Ly6C^hi^ inflammatory monocytes [21] or Cx3CR1^+^ progenitors [5], do not self-renew and have a short life span in the blood stream, we performed FNA one day before BMT (Figure 3A). HI was carried out 14 days prior to transplantation to match the peak of chemokine/cytokine expression after irradiation (cf. Figure 1B) with the time points of analysis at 7 and 14 days after BMT (i.e. 3 and 4 weeks after irradiation). For comparison, we also used the chemotherapeutic agent busulfan for conditioning (Figure 3A). Busulfan has recently been demonstrated to trigger the entry of BMDCs into the brain with reduced CNS inflammation [24]. Mice receiving either HI or FNA alone prior to BMT served as control groups (Figure 3A).

As expected given the myelotoxic properties of busulfan, HI treatment resulted in lower blood chimerism compared to busulfan conditioning (HI + FNA: 5 ± 0.7% versus busulfan + FNA: 50 ± 4%; Figure 3B). FNA had no impact on blood chimerism (HI + FNA: 5 ± 0.7% versus HI: 8 ± 1%; Figure 3B), and conditioning was required to establish blood chimerism (FNA: <0.5%; Figure 3B). Notably, the vast majority of GFP^+^ cells in peripheral blood of HI, HI + FNA and busulfan + FNA chimeras were identified as CD11b^+^ myeloid cells (> 90% of GFP^+^ cells; Figure 3C). These included Ly6C^hi^ monocytes (HI: 8 ± 1%; HI + FNA: 9 ± 1% and busulfan: 10 ± 2% of GFP^+^CD11b^+^ cells; Figure 3C) and Ly6G^+^ neutrophils (HI: 60 ± 3%; HI + FNA: 62 ± 2% and busulfan: 67 ± 7.0% of GFP^+^CD11b^+^ cells; Figure 3C). No significant differences in the contribution of donor-derived myeloid cells were observed between the groups. 

**Figure 4:**
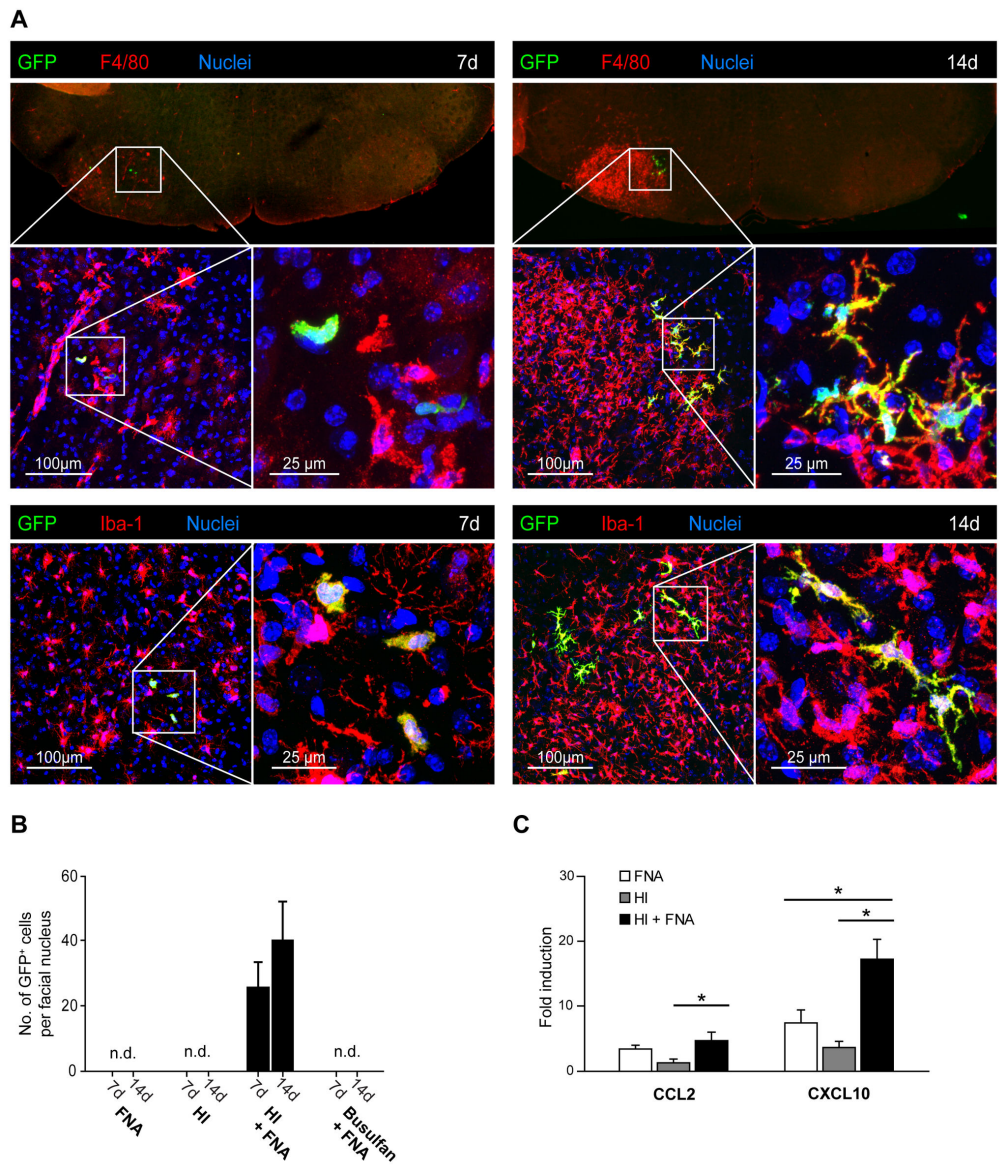
Selective engraftment of donor-derived myeloid cells in the lesioned facial nucleus after HI. **A**) Identification of GFP^+^ myeloid cells in the lesioned facial nucleus of HI chimeras at 7 and 14 days after BMT. Note the increase in F4/80 immunoreactivity at day 14 compared with day 7, indicating increased inflammation. The contralateral unlesioned facial nucleus is devoid of donor-derived GFP^+^ cells and shows minimal F4/80 immunoreactivity. Laser confocal microscopic images of areas of interest (white squares) are shown at increasing magnifications (scale bars: 100 µm – 25 µm). Seven days after BMT, amoeboid GFP^+^F4/80^+^ and GFP^+^Iba-1^+^ cells were detected in the lesioned facial nucleus. At 14 days after BMT, GFP^+^F4/80^+^ and GFP^+^Iba-1^+^ cells in the lesioned facial nucleus were highly ramified. All donor-derived GFP^+^ cells expressed the macrophage markers, F4/80 and Iba-1. Nuclei were counterstained with DAPI. **B**) Quantification of myeloid cell engraftment in the lesioned facial nucleus at 7 and 14 days after BMT in FNA, HI, HI + FNA and busulfan + FNA animals. Note that donor-derived GFP^+^ cells were only detected in HI + FNA mice. Data are means + SEM from 3-5 animals per group. n.d. = none detected. **C**) Quantitative real-time PCR of CXCL10 and CCL2 mRNA expression in the facial nucleus of animals with FNA (white bars), HI (grey bars) and HI + FNA (black bars) at 14 days after BMT. The mRNA expression levels were normalized to GAPDH mRNA and compared to naïve mice (fold induction). Increased chemokine mRNA levels were observed in the facial nucleus of HI + FNA mice compared to HI animals. Data are means + SEM from 3-5 animals per group. Statistical significance is indicated by asterisks (*p<0.05).

As early as 7 days after BMT, GFP^+^ cells were specifically detected in the lesioned facial nucleus of HI + FNA mice (26 ± 7 cells/facial nucleus; Figures 4A,B). These cells were amoeboid and localized in the brain parenchyma. They were immunoreactive for Iba-1 and F4/80, which are markers of macrophages (Figure 4A). At 14 days after BMT, GFP^+^ cells with a characteristic ramified morphology and expression of Iba-1 and F4/80 were detected in the lesioned facial nucleus of HI + FNA mice (40 ± 12 cells/nucleus, Figures 4A,B). The unlesioned contralateral facial nucleus was devoid of GFP^+^ cells at all time points (Figure 4A). Notably, no donor-derived GFP^+^ cells were observed in the lesioned facial nucleus of busulfan + FNA mice at 7 and 14 days after BMT despite high levels of blood chimerism (Figure 4B). Similarly, FNA and HI alone also failed to recruit donor-derived myeloid cells into the lesioned facial nucleus at 7 and 14 days after BMT (Figure 4B). This is in line with the delayed engraftment kinetics of myeloid cells after HI treatment (Figures 2B,C), and with published evidence documenting the necessity of conditioning for myeloid cell engraftment in the CNS [7,21].

Since myeloid cell recruitment into the CNS has been suggested to correlate with the induction of CCL2 and CXCL10 mRNAs [21], we used real-time PCR to quantify the expression levels of both chemokines in the facial nucleus of mice with FNA, HI and HI + FNA. The expression of CXCL10 and CCL2 mRNAs was potentiated in the HI + FNA group at 14 days after BMT (Figure 4C). The results suggest that HI and FNA act synergistically to induce chemokines, which may accelerate the recruitment of myeloid cells into the CNS. 

## Discussion

We established a novel non-myeloablative conditioning protocol for targeting BMDCs to the brain using focal head irradiation plus rapamycin. In contrast to TBI or busulfan treatment, this conditioning regimen minimally disturbs the host’s hematopoietic system and enables rapid transmigration of adoptively transferred myeloid cells into the CNS.

BMDCs have emerged as promising treatment vehicles in neurological and psychiatric disorders [6,14,18,19,22,23]. However, the transmigration of BMDCs into the brain is tightly regulated. In animal models, in which peripheral blood chimerism was obtained by parabiosis, no engraftment of BMDCs in the adult brain was observed [4]. Similary, no CNS engraftment of BMDCs was observed after irradiation with brain protection [4,21]. In contrast, TBI is a highly effective conditioning regimen for targeting BMDCs to the rodent brain [6,24,26]. 

In the past, TBI has played an important role in patients undergoing HSC transplantation. However, TBI may result in serious acute graft-versus-host disease (GVHD) causing transplant-related morbidity and mortality, as well as damage to non-target tissues, which may predispose to GVHD or enhance the clinical manifestations of acute GVHD [28-30]. Targeted irradiation with selective delivery of myeloablative doses to bone and marrow resulted in reduction of tissue damage and allowance for dose escalation compared with conventional TBI [28]. Thus, focal irradiation may serve as an alternative regimen for host conditioning that causes less irradiation/transplant-related morbidity and mortality. Furthermore, the availability of a computed tomography (CT) image-guided radiotherapy, such as helical tomotherapy, provides the opportunity to deliver highly conforming dose distributions to complex target shapes while simultaneously avoiding excessive doses to critical normal tissue [28]. 

In this study, we established a conditioning protocol in mice using targeted head irradiation that induced comparatively low inflammatory responses in the CNS despite cranial radiation doses which were identical to TBI. Moreover, HI minimally perturbed the host’s hematopoietic compartment. In contrast to TBI and busulfan chemotherapy, the HI regimen achieved very low chimerism in peripheral blood. Nevertheless, HI conditioning enabled rapid and selective recruitment of myeloid cells to sites of brain damage. Note that HI alone did not trigger any CNS engraftment of BMDCs in the absence of additional brain damage. Engraftment occurred with a short latency after BMT (7 days). In contrast, busulfan treatment failed to target myeloid cells to the CNS within 14 days after BMT. Although myeloablative chemotherapy with busulfan is often used clinically in allogeneic HSC transplantation, the effectiveness of busulfan conditioning in triggering BMDC recruitment to the rodent brain remains controversial. Lampron et al. did not observe any donor-derived cells in the CNS after the administration of 80 mg/kg of busulfan plus 200 mg/kg cyclophosphamide before BMT [26]. Other reports showed reduced [24,31] or enhanced [32] BMDC engraftment compared with TBI using 80 mg/kg, 90 mg/kg and 125 mg/kg of busulfan, respectively. In our study, the myelosuppressive dose of 100 mg/kg busulfan failed to rapidly target BMDCs to the CNS within the first two weeks after BMT.

In order to prevent transplant rejection, we administered the immunosuppressant drug, rapamycin, in the FNA, HI, HI+FNA and busulfan+FNA groups. Thus, the differences in CNS engraftment of BMDCs between the groups cannot be attributed to rapamycin. Moreover, rapamycin has been used to stabilize the blood-brain barrier [33]. 

Cytokines and chemokines are among the signals which may mediate the transmigration of BMDCs into the brain. Notably, TBI and busulfan treatment were found to induce CCL2, CXCL10, CCL3, CCL5, TNF-α and IL-1 gene expression in the murine brain [21,24,32]. We observed that HI induces the same pattern of cytokines/chemokines, but to a much lower degree than TBI. CCL2 is *de novo* expressed in facial motoneurons after axotomy [34]. Interestingly, HI increases CCL2 mRNA expression in the axotomized facial nucleus, which may trigger myeloid cell engraftment.

The receptor for CCL2, CCR2, was suggested to be necessary for the egression of monocytes from the bone marrow (BM) to the spleen [35] and from the blood stream to the tissue [36], including the brain [21]. Results obtained in chimeric mice generated by TBI and transplantation of CCR2-deficient BM cells suggested that BM-derived myeloid cells in the adult brain originate from circulating Ly6C^hi^CCR2^+^ inflammatory monocytes [21]. In contrast, using parabiotic mice, Ajami et al. showed that the progenitors of BM-derived myeloid cells in the brain do not spontaneously enter the blood stream, but need to be artificially administered into the circulation. The authors proposed HSCs and CX_3_CR1^+^ myelomonocytic progenitors (a mixture of non-self-renewing progenitor populations) as sources of microglia and brain macrophages, respectively [4,5]. However, these results were based on hematopoietic reconstitution of the TBI recipients by transplantation of partially purified BMDCs. Since selective transplantation of purified BMDC populations is not possible in a myeloablative setting, the precise characterization of the precursors of brain myeloid cells has posed a significant challenge. The optimized conditioning and transplantation regimen presented in this study allows for the first time to adoptively transfer selected BMDC populations into wild-type recipients and to perform short-term analysis of the transmigration of BMDCs into the CNS without hematopoietic reconstitution. Thus, the HI protocol provides a valuable tool for tracking the fate of short-lived BMDCs and for identifying the precursors of brain macrophages, a promising cell population for the treatment of neurodegenerative disorders. 

## References

[1] Ginhoux F, Greter M, Leboeuf M, Nandi S, See P et al. (2010) Fate mapping analysis reveals that adult microglia derive from primitive macrophages. Science 330: 841-845. doi:10.1126/science.1194637. PubMed: 20966214. 20966214 PMC3719181

[2] Schulz C, Gomez Perdiguero E, Chorro L, Szabo-Rogers H, Cagnard N et al. (2012) A lineage of myeloid cells independent of Myb and hematopoietic stem cells. Science 336: 86-90. doi:10.1126/science.1219179. PubMed: 22442384. 22442384

[3] Kierdorf K, Erny D, Goldmann T, Sander V, Schulz C et al. (2013) Microglia emerge from erythromyeloid precursors via Pu.1- and Irf8-dependent pathways. Nat Neurosci 16: 273-280. doi:10.1038/nn.3318. PubMed: 23334579. 23334579

[4] Ajami B, Bennett JL, Krieger C, Tetzlaff W, Rossi FMV (2007) Local self-renewal can sustain CNS microglia maintenance and function throughout adult life. Nat Neurosci 10: 1538-1543. doi:10.1038/nn2014. PubMed: 18026097. 18026097

[5] Ajami B, Bennett JL, Krieger C, McNagny KM, Rossi FMV (2011) Infiltrating monocytes trigger EAE progression, but do not contribute to the resident microglia pool. Nat Neurosci 14: 1142–1149. doi:10.1038/nn.2887. PubMed: 21804537. 21804537

[6] Priller J, Flügel A, Wehner T, Böntert M, Haas CA et al. (2001) Targeting gene-modified hematopoietic cells to the central nervous system: Use of green fluorescent protein uncovers microglial engraftment. Nat Med 7: 1356-1361. doi:10.1038/nm1201-1356. PubMed: 11726978. 11726978

[7] Hess DC, Abe T, Hill WD, Studdard AM, Carothers J et al. (2004) Hematopoietic origin of microglial and perivascular cells in brain. Exp Neurol 186: 134-144. doi:10.1016/j.expneurol.2003.11.005. PubMed: 15026252. 15026252

[8] Hickey WF, Kimura H (1988) Perivascular microglial cells of the CNS are bone marrow-derived and present antigen in vivo. Science 15: 290-292. PubMed: 3276004. 10.1126/science.3276004 3276004

[9] Takahashi K, Prinz M, Stagi M, Chechneva O, Neumann H (2007) TREM2-transduced myeloid precursors mediate nervous tissue debris clearance and Facilitate recovery in an animal model of multiple sclerosis. PLOS Med 4: 675-689. 10.1371/journal.pmed.0040124 PMC1851623 17425404

[10] Ye M, Wang XJ, Zhang YH, Lu GQ, Liang L et al. (2007) Transplantation of bone marrow stromal cells containing the neurturin gene in rat model of Parkinson’s disease. Brain Res 1142: 206-216. doi:10.1016/j.brainres.2006.12.061. PubMed: 17336273. 17336273

[11] Makar TK, Trisler D, Bever CT, Goolsby JE, Sura KT et al. (2008) Stem cell based delivery of IFN-β reduces relapses in experimental autoimmune encephalomyelitis. J Neuroimmunol 196: 67-81. doi:10.1016/j.jneuroim.2008.02.014. PubMed: 18471898. 18471898

[12] Zhang S, Zou Z, Jiang X, Xu R, Zhang W et al. (2008) The therapeutic effects of tyrosine hydroxylase gene transfected hematopoetic stem cells in a rat model of Parkinson´s disease. Cell Mol Neurobiol 28: 529-543. doi:10.1007/s10571-007-9191-8. PubMed: 17713852. 17713852 PMC11514996

[13] Malm TM, Koistinaho M, Pärepalo M, Vatanen T, Ooka A et al. (2005) Bone marrow-derived cells contribute to the recruitment of microglial cells in response to β-amyloid deposition in APP/PS1 double transgenic Alzheimer mice. Neurobiol Dis 18: 134-142. doi:10.1016/j.nbd.2004.09.009. PubMed: 15649704. 15649704

[14] Simard AR, Soulet D, Gowing G, Julien JP, Rivest S (2006) Bone marrow-derived microglia play a critical role in restricting senile plaque formation in Alzheimer's disease. Neuron 49: 489-502. doi:10.1016/j.neuron.2006.01.022. PubMed: 16476660. 16476660

[15] Solomon JN, Lewis CA, Ajami B, Corbel SY, Rossi FM et al. (2006) Origin and disruption of bone marrow-derived cells in the central nervous system in a mouse model of amyotrophic lateral sclerosis. Glia 53: 744–753. doi:10.1002/glia.20331. PubMed: 16518833. 16518833

[16] Priller J, Prinz M, Heikenwalder M, Zeller N, Schwarz P et al. (2006) Early and rapid engraftment of bone marrow-derived microglia in scrapie. J Neurosci 26: 11753–11762. doi:10.1523/JNEUROSCI.2275-06.2006. PubMed: 17093096. 17093096 PMC6674798

[17] Djukic M, Mildner A, Schmidt H, Czesnik D, Brück W et al. (2006) Circulating monocytes engraft in the brain, differentiate into microglia and contribute to the pathology following meningitis in mice. Brain 129: 2394-2403. doi:10.1093/brain/awl206. PubMed: 16891321. 16891321

[18] Shechter R, London A, Varol C, Raposo C, Cusimano M et al. (2009) Infiltrating blood-derived macrophages are vital cells playing an anti-inflammatory role in recovery from spinal cord injury in mice. PLOS Med 6: e1000113Available online at: 10.1371/journal.pmed.1000113. 19636355 PMC2707628

[19] Derecki NC, Cronk JC, Lu Z, Xu E, Abbott SB et al. (2012) Wild-type microglia arrest pathology in a mouse model of Rett syndrome. Nature 484: 105-109. doi:10.1038/nature10907. PubMed: 22425995. 22425995 PMC3321067

[20] Hoogerbrugge PM, Suzuki K, Suzuki K, Poorthuis BJ, Kobayashi T et al. (1988) Donor-derived cells in the central nervous system of twitcher mice after bone marrow transplantation. Science 239: 1035-1038. doi:10.1126/science.3278379. PubMed: 3278379. 3278379

[21] Mildner A, Schmidt H, Nitsche M, Merkler D, Hanisch UK et al. (2007) Microglia in the adult brain arise from Ly6C^hi^CCR2^+^ monocytes only under defined host conditions. Nat Neurosci 10: 1544-1553. doi:10.1038/nn2015. PubMed: 18026096. 18026096

[22] Corti S, Locatelli F, Donadoni C, Guglieri M, Papadimitriou D et al. (2004) Wild-type bone marrow cells ameliorate the phenotype of SOD1-G93A ALS mice and contribute to CNS, heart and skeletal muscle tissues. Brain 127: 2518-2532. doi:10.1093/brain/awh273. PubMed: 15469951. 15469951

[23] Keshet GI, Tolwani RJ, Trejo A, Kraft P, Doyonnas R et al. (2007) Increased host neuronal survival and motor function in BMT Parkinsonian mice: involvement of immunosuppression. J Comp Neurol 504: 690-701. doi:10.1002/cne.21483. PubMed: 17722033. 17722033

[24] Kierdorf K, Katzmarski N, Haas CA, Prinz M (2013) Bone marrow cell recruitment to the brain in the absence of irradiation or parabiosis bias. PLOS ONE 8: e58544. doi:10.1371/journal.pone.0058544. PubMed: 23526995. 23526995 PMC3592806

[25] Cartier N, Hacein-Bey-Abina S, Bartholomae CC, Veres G, Schmidt M et al. (2009) Hematopoietic stem cell gene therapy with a lentiviral vector in X-linked adrenoleukodystrophy. Science 326: 818-823. doi:10.1126/science.1171242. PubMed: 19892975. 19892975

[26] Lampron A, Lessard M, Rivest S (2012) Effects of myeloablation, peripheral chimerism, and whole-body irradiation on the entry of bone marrow-derived cells into the brain. Cell Transplant 21: 1149-1159. doi:10.3727/096368911X593154. PubMed: 21944997. 21944997

[27] Okabe M, Ikawa M, Kominami K, Nakanishi T, Nishimune Y (1997) 'Green mice' as a source of ubiquitous green cells. FEBS Lett 407: 313-319. doi:10.1016/S0014-5793(97)00313-X. PubMed: 9175875. 9175875

[28] Wong JY, Liu A, Schultheiss T, Popplewell L, Stein A et al. (2006) Targeted total marrow irradiation using three-dimensional image-guided tomographic intensity-modulated radiation therapy: an alternative to standard total body irradiation. Biol Blood Marrow Transplant 12: 306-315. doi:10.1016/j.bbmt.2005.10.026. PubMed: 16503500. 16503500

[29] Clift RA, Buckner CD, Appelbaum FR, Bearman SI, Petersen FB et al. (1990) Allogeneic marrow transplantation in patients with acute myeloid leukemia in first remission: a randomized trial of two irradiation regimens. Blood 76: 1867-1871. PubMed: 2224134. 2224134

[30] Clift RA, Buckner CD, Appelbaum FR, Bryant E, Bearman SI et al. (1991) Allogeneic marrow transplantation in patients with chronic myeloid leukemia in the chronic phase: a randomized trial of two irradiation regimens. Blood 77: 1660-1665. PubMed: 2015394. 2015394

[31] Lewis C-AB, Manning J, Barr C, Peake K, Humphries RK et al. (2013) Myelosuppressive conditioning using busulfan enables bone marrow cell accumulation in the spinal cord of a mouse model of amyotrophic lateral sclerosis. PLOS ONE 8: e60661. doi:10.1371/journal.pone.0060661. PubMed: 23593276. 23593276 PMC3620474

[32] Wilkinson FL, Sergijenko A, Langford-Smith KJ, Malinowska M, Wynn RF et al. (2013) Busulfan conditioning enhances engraftment of hematopoietic donor-derived cells in the brain compared with irradiation. Mol Ther 21: 868-876. doi:10.1038/mt.2013.29. PubMed: 23423338. 23423338 PMC3616529

[33] van Vliet EA, Forte G, Holtman L, den Burger JC, Sinjewel A et al. (2012) Inhibition of mammalian target of rapamycin reduces epileptogenesis and blood-brain barrier leakage but not microglia activation. Epilepsia 53: 1254-1263. doi:10.1111/j.1528-1167.2012.03513.x. PubMed: 22612226. 22612226

[34] Flügel A, Hager G, Horvat A, Spitzer C, Singer GM et al. (2001) Neuronal MCP-1 expression in response to remote nerve injury. J Cereb Blood Flow Metab 21: 69-76. PubMed: 11149670. 11149670 10.1097/00004647-200101000-00009

[35] Serbina NV, Pamer EG (2006) Monocyte emigration from bone marrow during bacterial infection requires signals mediated by chemokine receptor CCR2. Nat Immunol 7: 311–317. doi:10.1038/nrm1909. PubMed: 16462739. 16462739

[36] Swirski FK, Libby P, Aikawa E, Alcaide P, Luscinskas FW et al. (2007) Ly-6Chi monocytes dominate hypercholesterolemia-associated monocytosis and give rise to macrophages in atheromata. J Clin Invest 117: 195-205. doi:10.1172/JCI29950. PubMed: 17200719. 17200719 PMC1716211

